# Prevalence and Risk Factors of Bovine Mastitis in Southern Zone of Tigray, Northern Ethiopia

**DOI:** 10.1155/2021/8831117

**Published:** 2021-06-28

**Authors:** Adehanom Baraki, Teshale Teklue, Tesfay Atsbha, Temesgen Tesfay, Solomon Wayou

**Affiliations:** ^1^Mekelle Agricultural Research Center, P.O.Box 258, Mekelle, Ethiopia; ^2^Alamata Agricultural Research Center, P.O.Box 56, Alamata, Ethiopia; ^3^Raya University, P.O.Box 92, Maichew, Ethiopia

## Abstract

Mastitis is the most common disease of adult dairy cattle. The disease continues to be one of the most perplexing and costly diseases of dairy cattle. The present study was conducted to detect bovine mastitis cows and identify potential risk factors for the distribution of bovine mastitis of smallholder dairy farmers using the California mastitis test. A cross-sectional study was conducted from September 2013 to May 2015 in the southern zone of Tigray, Northern Ethiopia, on 146 head of randomly selected cattle, of which 99 (67.8%) were crossed zebu and 47 (32.2%) pure local zebu using the California mastitis test and questionnaire. The overall prevalence was 65 (45.5%), of which 72.3% of crossed local and 27.69% of local zebu were found positive for the CMT test. The investigated risk factors were the season of lactation (OR = 0.510, CI = 0.208–1.247, *P*=0.140), washing hands between consecutive milking events (OR = 0.354, CI = 0.146–0.856, *P*=0.021), and history of previous mastitis (OR = 0.441, CI = 0.226–0.858, *P*=0.016), which had significant (*P* < 0.05) effect on the present prevalence of bovine mastitis in the study area. A high prevalence of bovine mastitis was observed in the southern zone of Tigray. The summer season of lactation and not washing hands between consecutive milking of cows were significant mastitis causation risk factors in addition to the history of previous mastitis disease history.

## 1. Introduction

Dairy production is one of the livestock production sectors in many parts of Ethiopia [1], makes a major contribution to national and household economies, and provides milk for nutrition. Milk contributes significantly to meeting the human requirements for animal protein and is especially important in the diet of children and the sick [2]. Despite this, the national and regional milk supply was low due to lack of market awareness [1], lack of nutrients, and disease in animals, where mastitis was among the aforementioned constraints of production [3].

Mastitis is the most prevalent infectious disease of adult dairy cattle. Udder infection may develop when the cow is lactating or dry [4]. Hence, the role of milk in the transmission of disease, management practices and regulations to ensure safe dairy products reach consumers, current challenges facing the dairy industry and impacts on public health, and how these standards can be employed in low- and middle-income countries to improve public health, nutrition, and economic benefits to farmers have to be investigated and understood [5]. Mastitis continues to be one of the most perplexing and costly diseases of dairy cattle. Mastitis control relies upon the application of effective control measures to the herd rather than identification or special treatment of individual animals. It is essential to identify the organisms responsible for the problem and, in high-cell-count herds, the proportion of infected cows [4]. Most new infections occur during the early part of the dry period and in the first two months of lactation, especially with environmental pathogens. In heifers, the prevalence of infection is often high in the last trimester of pregnancy and several days before parturition, followed by a marked decline after parturition [6].

Mastitis is a major disease in dairy cattle in Ethiopia [7], and it was prioritized as one of the major diseases of dairy cattle. However, the risk factors of mastitis in the southern zone of the Tigray region, Ethiopia, were not investigated before.

This study aimed to assess the risk factors for the distribution of the disease and to determine the level of distribution of the disease in the study area.

## 2. Materials and Methods

### 2.1. Study Area

**Figure 1:**
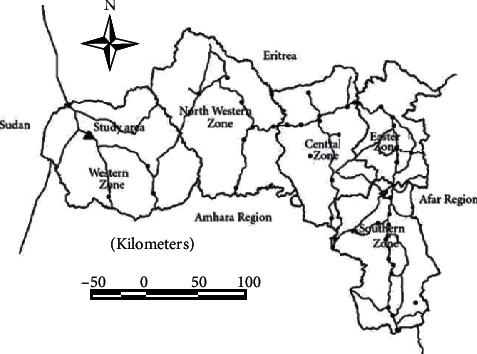
Map of Tigrai regional state showing the study area [8].

Tigray region: the Tigray Regional State is located in the northern part of Ethiopia, with a total area of approximately 102,000 square kilometers. The region has a common boundary with Eritrea in the north, Sudan in the west, Amhara Regional State in the south, and Afar Regional State in the east. It extends 12° 13′ to 14° 54′ north latitude and 36° 27′ to 40° 18′ east longitudes. Elevation ranges from 500 to 3900 meters above sea level. Mean annual rainfall was less than 200 mm in eastern escarpment to over 1900 mm in the southwestern part of the region. The main rainy season is summer between June and September, with short spring rain in the south and autumn rains in the eastern part of the region. Livestock and crop husbandry play a considerable role in the subsistence farming of Tigray. The majority of the population, above 90% of the total, was dependent on this ancient form of plough based-cultivation. Subsistence farming has been practiced in the region without showing improvement. Limited mechanized farming is being practiced in western lowlands [8]. The administrative location and boundaries of the southern zone of Tigray are shown in Figure 1.

According to the Central Statistical Agency of Ethiopia [9] survey, Tigray region possesses 4,817, 104 head of cattle, 2,472,938 head of sheep, 4,301,221 goats, 15,664 horses, 11,308 mules, 886,103, 43, 332 camels, 6,190,640 poultry, and 293,184 bee colonies accounting for 9.52 percent of the country's total livestock population. The region presently offers the global market a wide range of processed and semiprocessed hides and skins. Some of the products, such as highland sheepskin, are renowned for their quality and natural characteristics. The export of finished leather and leather products (such as leather garments, footwear, gloves, bags, and other leather articles) is also increasing. Representative areas of the region were selected based on agroecology, livestock population, and border with other regions.

### 2.2. Study Animals

The study animals were lactating dairy cattle that are managed under both the extensive and semi-intensive system. The dairy cattle under study comprised the local breeds and cross breeds of cattle.

### 2.3. Study Design

A cross-sectional study was carried out to determine the overall individual animal and herd level prevalence of mastitis, etiologies, and associated risk factors. During the beginning of the study, community identification and assessment complete list of dairy herds were made. A questionnaire was conducted at the same time after herds and flock owners were identified.

### 2.4. Herd Selection and Sampling

Multistage sampling methods were followed to select districts, peasant associations, herds, and animals as the primary, secondary, tertiary, and quarterly units, respectively. At each stage, sampling units were selected randomly [10]. From each district, 10% of peasant associations were selected. From each peasant association, 10% of the dairy herds were sampled. The number of animals selected from each herd was 10% of the total animals within the herd. The minimum sample size consisted of 146 lactating cows either at early part of the dry period from different breeds according to [11], with the expectation of 50% prevalence as in most countries, the prevalence of mastitis is 50% [6]. However, due to lack of evidence, 146 head of cattle were sampled.

### 2.5. Data Collection

#### 2.5.1. Questionnaire Survey

A pretested semistructured questionnaire was developed for information gathering on factors influencing the spread of mastitis infection within or between herds by face-to-face interviews with farmers while conducting screening tests. Data on breed, sex, age, herd size, previous mastitis history, animal management, farm grazing type, watering points, and agroecology of the area were recorded. Moreover, data on the habit of washing udder, disinfecting milking material, and hand washing practice before and after milking of individual cow were recorded.

#### 2.5.2. Screening

California mastitis test (CMT): the California mastitis test (CMT) was used to detect subclinical mastitis. The procedure and the interpretations were according to [12]. The results read as negative (0), trace (T), weak positive (+), distinct positive (++), and strong positive (+++). Reactions that included weak positive and above were considered as indicators of subclinical mastitis.

  Test procedure
(i) Collect milk into the CMT paddle.
(ii) After discarding the first stream of milk, draw the next milk into shallow cups on the paddle, keeping the quarters separate.
(iii) Always assume the same position when holding the paddle under the udder to keep track of the quarters when interpreting results.
(iv) Drain excess milk.
(v) The ideal amount of milk is that which remains in the cup when the paddle is tilted to an almost vertical position.
(vi) Add an equal amount of the reacting solution.
(vii) Form pools of milk in cups by tilting paddle.
(viii) Squirt test solution over milk. Avoid making bubbles.
(ix) Proportion of solution to milk should be at least one to one. Mix the reagent and milk.
(x) Gently rotate the paddle in the horizontal plane, swirling the mixture for 10–30 seconds.
(xi) Positive reactions occur and can be graded during this rotary motion.

### 2.6. Data Analysis

The collected data were filled on an Excel spreadsheet and data were analyzed using SPPSS version 20. Descriptive statistics was used to show the frequency of occurrence; chi-square test was used in testing the strength of association for mastitis causation; then, those showing strong associations were computed using odds ratio.

## 3. Results

### 3.1. Socioeconomic Characteristics and Dairy Constraints

A total of 146 respondents participated in the questionnaire, in which 126 (86.3%) and 20 (13.7%) were males and females, respectively, and their corresponding age groups were young age (14, 9.6%), middle age (64, 43.8%), and adult age (68, 46.6%). The level of education was also studied, in which illiterates were 43 (29.5) and those with elementary education level were 47 (32.2%), high school education 13 (8.9%), and highest education level 43 (29.5%). Factors affecting the dairy farm production and expansion were listed by dairy cow producers accordingly: feed shortage (76), land shortage (45), artificial insemination (AI) (8). Disease, market, and lack of credit are among the listed constraints in order of ascending importance in which the number indicates the number of respondents ranking these constraints as the number one major problem. The respondents were asked if they had dairy-related training; accordingly, 86 respondents (58.9%) said that they were trained and 60 (41.09%) were not trained. Respondents were also asked to list the possible causes of mastitis; accordingly, sanitation problems showed good results with 25 respondents, of which 12 (48%) dairy cow producers found mastitis and mechanical or traumatic damage, hyponutrition, and tick infestation among the raised mastitis risk factors.

### 3.2. Association of Different Factors for Mastitis Causation

Around 146 dairy cows were examined from all districts of the zone using the CMT (California mastitis test) screening test, in which 65 (44.52%) cows and 122 (20.89%) quarter teats were found positive and 6 (4.1%) cows had blind teat. The season of lactation period (*P*=0.012), history of herd mastitis presence (*P*=0.015), and washing hands during milking (*P*=0.018) were found to be significant risk factors for mastitis causation, in which summer or rainy season, history of udder illness, and not washing between milking events showed higher mastitis prevalence, respectively. The other possible risk factors and their level of significance are shown in Table 1. Significant associative risk factors affecting the prevalence of bovine mastitis are indicated in Table 2.

## 4. Discussion

From a total of 146 head of cows, the overall mastitis prevalence in the study area was 65 (45.5%) cows and 122 (20.89%) quarter teats and 6 (4.1%) cows were found to have blind teats using the CMT test. These results were a little different from the finding of [13], in which the prevalence was 56.5%, quarter level 31.4%, and blind level 10.6% in Batu, Ethiopia. This is due to sample size and breed differences across different study areas. The prevalence of blind teat (4.6%) agrees with the finding of [14]. Prevalence at cow level was significantly lower than the finding of [15] in Holeta town, which was 71.0%. This was due to the management difference. The current mastitis prevalence result also disagrees with the finding of [16] in Bahir Dar town, which was low (28.1%).

Factors affecting the prevalence of mastitis include lactation stage, season of calving, parity of animals, age of animals, breed of animals, hygiene of udder and teat, towel usage, and floor type. According to the current results, the history of herd mastitis, season of lactation, and washing hand before and b/*n* milking were found significant factors for causing mastitis (*P* < 0.05). However, the other predetermined listed factors have got no significant association. The results of the lactation stage across local breeds were similar to those of [16], where no significant associations were observed, but the results of the parity of animals contradicted with [16]. The hygienic udder practice was found to be a significant risk factor, which is similar to the finding of [15]. The lactation stage, parity, and breed results contradicted the finding of [17] in Hawassa because the management factors were more influential. The results of age and parity showed statistically insignificant (*P* > 0.05) association, agreeing with the finding of [13]. In the current study, the habit of using towels and lactation stage had an insignificant (*P* > 0.05) association with mastitis causation, agreeing with the finding of [14]. However, the results regarding previous mastitis history disagreed with this finding, showing a significant association (*P* < 5) in the current study, due to the chronic nature of the disease manifests for recurrent occurrence.

Generally, in this study, possible knowledge gaps were identified in dairy farmers regarding the occurrence of mastitis and its possible risk factors. From the respondents, about 21.2% wash their hands between consecutive milking of cows and around 75.8% did not use a towel to clean the udder, which agrees with several findings [14, 18, 19]. When milk is consumed unpasteurized, as it often occurs in developing countries where regulation and oversight of the dairy industry are lacking, dairy can serve as a vector for zoonotic transmission of disease and can contain adulterants such as antibiotic residues [5]. Mastitis milk and other dairy products are reported to be frequently infected with enterotoxin-producing *Staphylococcus aureus* [20]. Also, *Streptococcus agalactiae* has been described as one of the most common agents of invasive infections and public health hazards. So, those dairy producers understood the premise that food safety and milk quality begin on the farm [21].

## 5. Conclusions

In conclusion, the current study shows a high prevalence of bovine mastitis observed in the southern zone of Tigray. The summer season of lactation and not washing between consecutive milking of cows were significant mastitis causation risk factors, in addition to the history of previous mastitis disease history. However, in the present study, age, breed, lactation stage, and parity were not significant risk factors. Furthermore, possible knowledge gaps were identified in dairy farmers regarding the occurrence of mastitis and its possible risk factors.

## Figures and Tables

**Table 1:** Proportion of associative risk factors and level of significance.

Variables	Category	CMT result		Total positives (%)	Significance level (*P*=0.05)		
		Negative	Positive		*X* ^2^ value	DF	*P* value
*Districts*	Raya Alamata	33 (56.9%)	25 (43.1%)	39.7			
	Endamehoni	10 (41.7%)	14 (58.3%)	16.4			
	Emba Alaje	12 (46.2%)	14 (53.8%)	17.8			
	Raya Azebo	17 (63%)	10 (37%)	18.5	6.518^a^	4	0.164
	Ofla	9 (81.8%)	2 (18.2%)	7.5			
	Total prevalence	81 (55.5%)	65 (44.5%)	44.5			

*Agroecology*	Lowland	33 (56.9%)	25 (43.1%)	39.7			
	Highland	31 (50.8%)	30 (49.2%)	41.8	1.196^a^	2	0.550
	Mid highland	17 (63%)	10 (37%)	18.5			

*Season of lactation*	Autumn	17 (58.6%)	12 (41.1%)	19.9			
	Winter	38 (69.1%)	17 (30.9%)	37.7	8.846^a^	2	0.012
	Summer	26 (41.9%)	36 (58.1%)	42.5			

*History of herd mastitis*	Yes	31 (44.9	38 (55.1)	47.3	5.898^a^	1	0.015
	No	50 (64.9)	27 (35.1)	52.7			

*Breed examined*	Local	28 (59.6%)	19 (40.4%)	32.2	0.471^a^		0.493
	Cross	53 (53.5%)	46 (46.5%)	67.8		1	

*Production system*	Intensive	51 (56%)	40 (44%)	63.2			
	Extensive	23 (62.2%)	14 (37.8%)	25.3	2.687^a^	2	0.261
	Semi-intensive	7 (38.9%)	11 (61.1%)	12.3			

*Floor type*	Dust		40 (50.6%) 39 (49.4%)	54.1	1.637^a^	1	0.243
	Concrete	41 (61.2%)	26 (38.8)	45.9			

*Washing during milking*	Before and after	23 (74.2%)	8 (25.8%)	21.2	5.580^a^	1	0.018
	Before milking only	58 (50.4%)	57 (49.6%)	78.8			

*Towel usage parity*	Yes	22 (61.1%)	14 (38.9%)	24.7	0.614^a^	1	0.433
	No	59 (53.6%)	51 (46.4%)	75.3			
	Heifers (≤2)	41 (54.7%)	34 (45.3%)	52.3			
	Middle (3–5)	34 (54.8%)	28 (45.2%)	43.1	0.486^a^	2	0.784
	Multiparous (≥6)	6 (66.7%)	3 (33.3%)	4.6			

*Age of animals*	Heifers	25 (54.3%)	21 (45.7%)	31.5			
	Middle age	39 (60%)	26 (40%)	44.5	1.238^a^	2	0.539
	Old age	17 (48.6%)	18 (51.4%)	24			

*Lactation stage*	Early (≤3 months)	30 (61.2%)	19 (38.8%)	33.6			
	Middle (4–6)	32 (62.7%)	19 (37.3%)	34.9	5.487^a^	2	0.064
	Late (≥7)	19 (41.3%)	27 (58.7%)	31.5			

**Table 2:** Significant associative risk factors affecting the prevalence of bovine mastitis on 95% confidence interval.

Risk factors	Category	*N*	Positives	OR	95% CI	*P* value
*Season*	Dry season	62	36	—	—	—
	Autumn	55	17	0.510	0.208–1.247	0.140
	Rainy season	29	12	0.323	0.151–0.693	0.04

*Mastitis history*	Yes	69	38	0.441	0.226 0.858-	0.016
	No	77	27	—	—	—

*Washing hand*	Before milking only	115	57	0.354	0.146 -	0.021
					0.856	

*Frequency*	b/n consecutive cows	31	8	—	—	

## Data Availability

Data used to support the findings of this study are available from the corresponding author upon request via email, adehanommek@gmail.com.

## Abbreviations

CMT: California mastitis test
CI: Confidence interval
OR: Odds ratio
CSA: Central Statistical Agency
DF: Degree of freedom.
